# Effects of virtual target size, position, and parallax on vergence-accommodation conflict as estimated by actual gaze

**DOI:** 10.1038/s41598-022-24450-9

**Published:** 2022-11-22

**Authors:** Chiuhsiang Joe Lin, Susmitha Canny

**Affiliations:** Department of Industrial Management, National Taiwan University of Science and Technology, Taipei, 10607 Taiwan, ROC

**Keywords:** Human behaviour, Risk factors, Eye manifestations, Fatigue

## Abstract

Due to the increased utilization of stereoscopic displays, the scope of the vergence–accommodation conflict has been studied extensively to reveal how the human visual system operates. The purpose of this work was to study the phenomenon of vergence–accommodation conflict by comparing the theoretical eye vergence angle (vergence response) and gaze-based eye vergence angle (vergence stimulus) based on eye tracker gaze data. The results indicated that the gaze-based eye vergence angle was largest at the greatest parallax. The result also revealed that the eye vergence angle accuracy was significantly highest at the nearest parallax. Generally, accuracy improves when virtual objects are put in the middle and close to participants' positions. Moreover, the signed error decreases significantly when the virtual object is in the middle. Based on the results of this study, we can gain a greater understanding of the vergence–accommodation conflict in the stereoscopic environment.

## Introduction

Virtual reality (VR) and augmented reality (AR) have long been influential in the collective imagination^1^. Virtual reality (VR) headsets are becoming more affordable to a wider population, including adults and children. They have been transformed from expensive devices requiring expertise and extensive set-ups to inexpensive and easy-to-use devices with a rapidly growing market for technology and application^2,3^. Despite significant advances in image display, image generation, post-processing, and capture techniques, the current and future quality of stereoscopic 3D technology is still viewed with scepticism by many consumers. The stereoscopic 3D effect should not be distracting because consumers prioritize naturalness, convenience, and appearance. However, it is challenging to generate stereoscopic 3D images from two good images^4^. Furthermore, immersion in virtual environments (VEs) for an extended period can result in symptoms of visual fatigue, including headaches, nausea, eye strain, diplopia, and dizzindess^2,5–7^.

Humans have the ability to form mental images. This ability allows humans to increase their focus on various objects in the environment they are observing^8^. However, if the object is simply a series of images displayed on a flat screen, the eye will easily become confused or lose track of the object's point of interest. This phenomenon occurs due to unexpected changes in an object's location or camera angle. In this case, the change in disparity eliminates binocular vision, making a confusing double image (diplopia) appear. It results from the eye's response to changes in depth, which affect its vergence and accommodation. Vergence and accommodation generally work together to produce sharp images. A negative feedback mechanism keeps them roughly in sync and keeps them controlled.

Nonetheless, immersion in a virtual environment leads to a conflict between vergence and accommodation, also known as a vergence–accommodation conflict (VAC)^9^. The conflict arises when 3D objects are shown on flat displays. The 3D display provides depth cues through the simulated scene, including occlusion, shading, size, and binocular disparity^6,9^. In contrast, a flat display is associated with cues of focus and blurring of objects on the retina, resulting in a conflict between vergence and accommodation^9^. This condition presents a challenge in developing stereoscopic 3D.

Many studies have been conducted to investigate the vergence–accommodation conflict in the visual system, especially the vergence eye movement system, by measuring changes in vergence and accommodation^9,10^. Vergence angles have been compared in matching (theoretical) and conflicting (actual) viewing conditions using ocular biomechanics and eye-tracking techniques^2^. A complex model of the eyes–head–neck and a biomechanics model of the eyes are required to simulate eye–head coordination. However, this model can be implemented only in a sophisticated device when the eye tracker is embedded in an interactive virtual reality setup, such as a head-mounted display. To conduct this study, we used eye-tracking, 3D stereoscopic displays, and trigonometric computations.

The eye tracker collected eye gaze data, which were used to calculate the eye vergence angle. In this case, additional computations were performed to calculate the vergence angles from raw eye tracker data because the vergence angles were not supplied in the eye tracker output. Thus, if users experienced difficulty recognizing the correct depth with their eyes or maintaining a constant focus on an object, their eye-gaze interaction performance would suffer, resulting in increased visual fatigue and frustration. Therefore, we focused on the virtual object's parallax, size, and position in the vergence response. This study can serve as a starting point for other studies on the vergence–accommodation conflict in virtual environments.

## Results

This study was conducted to investigate and compare theoretical vergence angle (response vergence) with gaze-based vergence angle (stimulus response)^11^. In addition, it investigates whether the parallax, size, and position of the virtual object can affect eye vergence angle. The accuracy and signed error of the theoretical eye vergence angle will be compared with the gaze-based eye vergence angle. Three parallax levels of the stereoscopic environment are manipulated (on the screen, 30 cm in front of the screen, and 60 cm in front of the screen), three sizes are used: 1.9 cm (small), 2.9 cm (medium), and 3.9 cm (large). Participants will see four balls appear in four different positions: top middle, top right, middle right, and middle. Based on the eye tracking data, we develop an equation based on trigonometric computation that can measure vergence angle. In this study, the main objective is to provide a better understanding of vergence-accommodation conflict in the stereoscopic environment.

The Tobii eye-tracker captured the participant's eye movement using a framerate at 60 Hz. One participant has around 14334 gaze data in timestamp 36 experiment combinations, and these are three types of data: fixation, saccade, and unclassified. In this study, we only use fixation point type coordinates to calculate the eye vergence angle. After the filter process is carried out, the remaining data is 90 percent of the total data exported from the eye tracker. As a result of the filter, tracked data can be reduced from 14334 to 12904. This section presents the results of a one-way repeated measures ANOVA for each dependent variable's three levels of parallax: eye vergence angle, accuracy, and signed error. When the ANOVA results revealed that there were significant effects, post hoc tests were performed using Tukey's HSD (*p* = 0.05).

**Figure 1:**
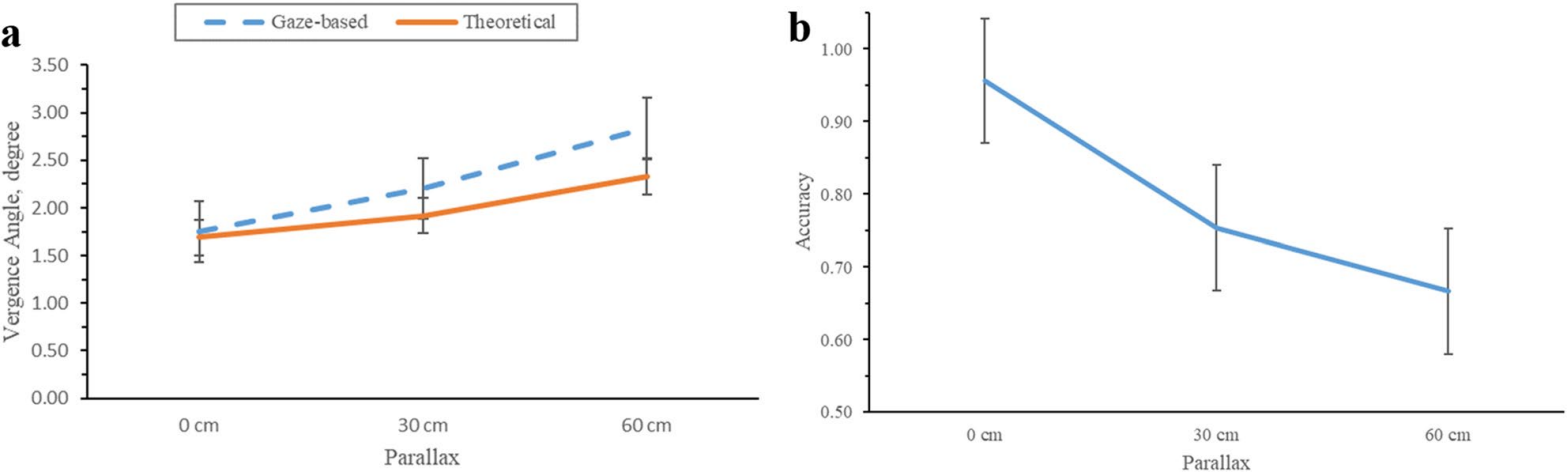
(**a**) Eye vergence angle based on gaze point and theoretical data concerning parallax, (**b**) Accuracy concerning parallax. The error bar shows the standard error of the mean.

The repeated measures ANOVA (Table 1) revealed that parallax significantly affected the gaze-based vergence angle (F_(2,22)_ = 27.043, *p* < .001). The overall result revealed that the average vergence angles based on the gaze point from the eye tracker were 1.753 degrees (SD = 0.083), 2.202 degrees (SD = 0.500), and 2.841 degrees (SD = 0.931) for 0, 30 cm, and 60 cm parallax, respectively. However, the theoretical vergence angles were 1.690 degrees (SD = 0.06), 1.917 degrees (SD = 0.08), and 2.327 degrees (SD = 0.127) for 0, 30 cm, and 60 cm parallax, respectively. It can be observed that in each parallax, the gaze-based vergence angle was larger than the theoretical vergence angle (See Fig. 1a). All pair-wise differences were significant from the grouping information, as determined by the Tukey method.

**Table 1:** Summary of repeated measures ANOVA results for gaze-based vergence angle. ***Note:*** Non-significant interactions are omitted from the ANOVA table.

Source	*F-value*	*p-value*
Parallax	27.043_(2, 22)_	< .001
Size	.632_(2, 22)_	.541
Position	2.015_(3, 33)_	.131
Parallax*Size*Position	2.549_(12,132)_	.005

The repeated measures ANOVA (Table 2) revealed that parallax (F_(2,22)_ = 22.006, *p* < 0.001) and position (F_(3, 33)_ = 3.954, *p* = 0.016) significantly affected the vergence angle accuracy. This result indicated that the overall accuracies of the vergence angles were 0.955 (SD = 0.014), 0.754 (SD = 0.193), and 0.666 (SD = 0.303) at zero, 30 cm, and 60 cm parallax, respectively. As shown in Fig. 1b., when the targets were displayed at the nearest parallax of 0 cm from the screen, the mean accuracy of the vergence angle increased significantly as compared to those of 30 cm and 60 cm parallax. Tukey post-hoc comparisons confirmed this observation, revealing that the mean score ($\mathrm{M}\pm \mathrm{SD})$ for zero parallax (0.955 $\pm$ 0.014) was significantly different from those of 30 cm (0.754 $\pm$0.193) and 60 cm parallax (0.666 $\pm$ 0.303).

**Table 2:** Summary of repeated measures ANOVA results for accuracy of eye vergence angle. ***Note:*** Non-significant interactions are omitted from the ANOVA table.

Source	F-*value*	*p-value*
Parallax	22.006_(2,22)_	< .001
Size	2.596_(1.370,15.074)_	.120
Position	3.954_(3,33)_	.016
Size*Position	2.561_(6,66)_	.027
Parallax*Size*Position	2.130_(12,132)_	.019

**Figure 2:**
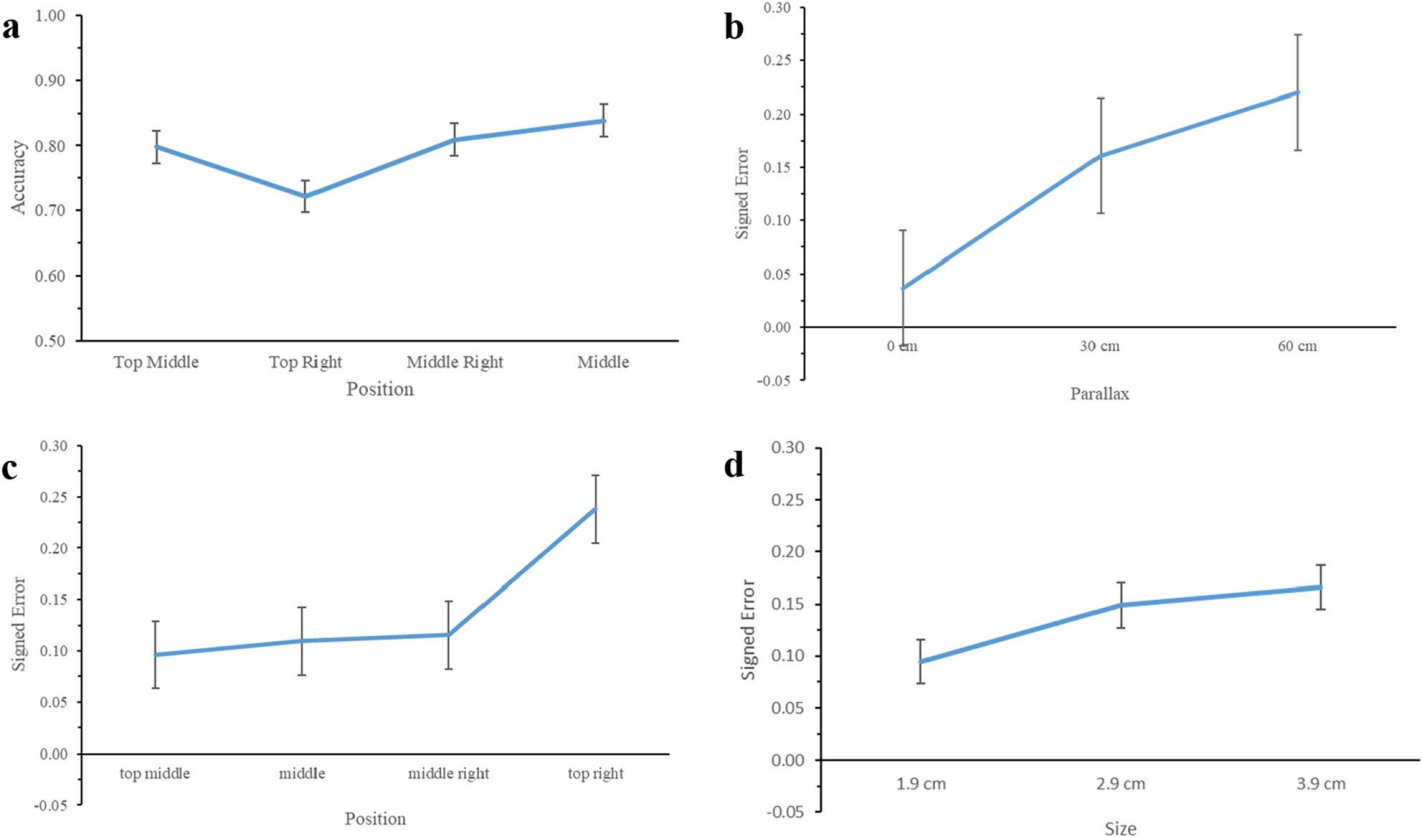
(**a**) Accuracy with respect to position, (**b**) Signed error with respect to parallax, (**c**) Signed error with respect to size, (d) signed error with respect to position. The error bar shows the standard error of the mean.

The average accuracy ($\mathrm{M}\pm \mathrm{SD})$ of the vergence angle at the middle (0.839 $\pm$ 0.206) was significantly higher than those of the middle right (0.809 $\pm$0.170), top middle (0.798 $\pm$0.236), and top right ($.$ 722 $\pm$ 0.312). It can be concluded that in the middle position had the highest accuracy (Fig. 2a.). In addition to the accuracy of the vergence angle, according to Tukey’s post hoc analysis, there were two groups of independent variables. Using a family error rate of 0.05, the results showed that the accuracy of the vergence angle was statistically significant at the 3 – 2 (middle right – top right) (*p* = 0.035) and 4 – 2 (middle right – top right) (*p* = 0.002) positions.

The repeated measures ANOVA (Table 3) revealed that parallax (F_(1.233, 13.558)_ = 4.503, *p* = 0.046), size (F_(2, 22)_ = 4.846, *p* = 0.018), and position (F_(3, 33)_ = 5.799, *p* = 0.003) significantly affected the signed error. The overall results revealed that the average signed errors were 0.037 ($\mathrm{SD}$ = 0.003), 0.152 ($\mathrm{SD}$ = 0.273), and 0.220 ($\mathrm{SD}$ = 0.394) for 0, 30 cm, and 60 cm parallax, respectively (shown in Fig. 2b.). It can be observed that the signed error was larger when the parallax increased. The Tukey HSD test indicated that that the signed error was significantly different between 30 cm and 0 parallax (*p* = 0.001) and between 60 cm and 0 parallax (*p* < 0.001).

**Table 3:** Summary of repeated measures ANOVA results for signed error of eye vergence angle. ***Note:*** Non-significant interactions are omitted from the ANOVA table.

Source	F-*value*	*p-value*
Parallax	4.503_(1.233, 13.558)_	.046
Size	4.846_(2, 22)_	.018
Position	5.799_(3, 33)_	.003
Parallax*Size	3.933_(4, 44)_	.008
Parallax*Size*Position	2.596_(12, 132)_	.004

The average signed error ($\mathrm{M}\pm \mathrm{SD})$ was significantly larger at 3.9 cm (0.166 $\pm$ 0.323) than at 2.9 cm (0.149 $\pm$0.259), followed by 1.9 cm (0.095 $\pm$ 0.272). The average signed error of the vergence angle was significantly larger at the top right (0.238 $\pm$ 0.334) than at the middle right (0.113 $\pm$ 0.230), followed by the middle (0.110 $\pm$ 0.238) and top right (0.085 $\pm$ 0.300) (See Fig. 2c). As illustrated in Fig. 2d, it can be concluded that the larger the size, the larger the signed error. In addition to the signed error of the vergence angle, the independent variables were categorized into two groups by Tukey's post hoc analysis. Using a family error rate of 0.05, the results showed that the accuracy of the vergence angle was statistically significant at the middle right – top right (*p* = 0.035) and middle – top right (*p* = 0.002) positions.

The results of repeated ANOVA revealed some significant interactions between the main factors discussed thus far. As shown in Table 1, analysis of the gaze-based vergence angles revealed significant interactions among parallax, size, and position (F_(12, 132)_ = 2.549, *p* = .005). As shown in Table 2, accuracy had an interaction between size and position (F_(6, 66)_ = 2.561, *p* = .027) and all other factor interactions (F_(12, 132)_ = 2.130, *p* = .019) were significant. Based on the repeated measure results in Table 3, signed error had an interaction between parallax and size (F_(4, 44)_ = 3.933, *p* = .008), and the interaction of three factors at the same time (F_(12, 132)_ = 2.596, *p*=.004) showed significant interactions.

## Discussion

According to the findings of this study, the gaze-based vergence angle was overestimated as compared with the theoretical vergence angle. This indicates that the eye condition is overestimating convergence. This indication reveals the conflict between vergence and accommodation, where constant accommodation due to a lack of blurry cues conflicts with vergence movement induced by changes in simulated depth in a virtual 3D space ^9,10^. The results of this study are consistent with those of previous studies^12,13^. Those studies discovered that space compression occurs in all three dimensions in virtual environments. When compression occurs, it affects the position of coordinates and makes the virtual object look smaller, making the object seem closer. In the virtual environment, the closer the object is, the smaller the size; however, the closer it is, the greater the angle formed. Furthermore, results from gaze-based data revealed that participants tended to overestimate the vergence angle. At 0, 30, and 60 cm from the screen (parallax), the vergence angle was overestimated by 1.75, 2.20, and 2.84 degrees, respectively. However, overestimation was reported in the majority of virtual vergence angle studies ^2,14^.

The significant relationship between simulated parallax and eye gaze points was discovered, and it impacted the vergence angle measurements. As the simulated parallax increased, participants found it difficult to maintain their fixation on the virtual object. As a result, we can conclude that increased simulated parallax impairs participants' ability to fix their gaze position on the virtual object. This condition implies increased visual fatigue in the participant's eyes. It was also discovered that the vergence angle at zero parallax improved. We also discovered that as parallax increased, the accuracy of the vergence angle decreased. This finding is consistent with previous research^12,13^, which found that the accuracy of the vergence angles decreases as virtual objects approach the eye. This finding is supported by the occurrence of conflict between vergence and accommodation^9^. A virtual object displayed closer to a participant produces greater vergence–accommodation mismatch than does a virtual object displayed farther away. This reduces the accuracy of the eye vergence angle in virtual environments.

Furthermore, it was discovered that virtual objects presented at a distance of 60 cm from the screen had the greatest error in terms of eye vergence angle. This result indicated that the signed error of eye vergence angle increases with a parallax (a virtual object's distance from the screen). This finding could lead to a better understanding of the appropriate space required for interaction with virtual objects, a closer relationship between interaction distances with virtual objects, and a reduction in fatigue caused by the display.

The effect of changing size on the vergence angle of signed error increased when the virtual object was larger. This finding is supported by Regan and Erkelens'^15^ research, which found that changing the size of virtual objects affected vergence, albeit only slightly. When the size of a virtual object increases, the area of the participant's field of view occupied by the object becomes wider than when the size of the virtual object decreases. As a result, the eye tracker will record various gaze positions. The signed error increases in size as the virtual object gets larger.

The results of four different position comparisons revealed that accuracy in the middle position had the most significant value. Furthermore, we discovered that the average value of the signed error in the top middle position had the smallest value based on the signed error value. These findings are consistent with previous research. Woldegiorgis and Lin^13^ discovered that participants have more difficulty judging the correct vertical (y) position when virtual objects are displayed at the bottom. The results of this study's experiment revealed that virtual objects on the right side of the display were affected more in the horizontal (x) position than virtual objects in the center. The overall distance evaluation in the stereoscopic environment improved when the virtual object was placed close to the center of the display. Based on the performance of the eye tracker, it is possible that the judgment of directionality in the virtual environment is the result of a systemic effect; looking up and to the right (dextroelevation) can affect both pupil size and the accuracy of the tracking. Another possible factor is the infrared effect of the 3D glasses emitter, which may have interfered with the Tobii eye tracker's infrared light. It can be regarded as a warning that simultaneous use of these instruments may lead to apparent deviations.

The conflict between vergence and accommodation is a major cause of visual fatigue. This study shows that it can impact our ocular system by causing excessive vergence eye movements, in which the vergence speed does not decrease and does not stabilize when our eyes converge to a specific parallax. However, the vergence angle has a higher median value when immersion occurs, indicating that the depth is perceived differently. This leads to incorrect depth perception and makes it difficult to fix one’s vision on objects at various depths.

This study used static targets with three parallax levels, three size levels, and four positions. In the future, further studies may need to be conducted to provide clarifications and more explanations about the effect of the height of the virtual object from the subject's eye on the vergence angle result. Consequently, Future research needs to adjust the trigonometric computation by adding an h variable (the height difference between objects and eyes). This may reveal more information about the interrelationship of some factors that affect the vergence-accommodation conflict. Different virtual environment systems and sensors can also be used in future research, such as head-mounted displays or devices that can work with mixed reality systems, which may include optical and inertial sensors to track orientation and position. The problem of the infrared signal interference or distraction may be avoided by using these devices.

## Methods

The main objective of this study was to provide further understanding of the vergence–accommodation conflict in a stereoscopic environment. To better understand this conflict, the accuracy and signed error of theoretical vergence angles were compared with gaze-based vergence angles.

### Participants

The participants were 12 graduate students from the National Taiwan University of Science and Technology, eight females and four males, ranging in age from 22 to 31 years old ($\mathrm{M}\pm \mathrm{SD}$= 24.5 $\pm$ 3.0). All participants had normal or corrected to normal visual acuity in this study. A stereo vision test was administered as a selection criterion to check each participant's maximum stereo vision. The participants received no pay, credit for their work, or any other form of compensation.

### Ethical statements

Before the experiment began, all participants provided informed consent to the publication of their identifying information/images and their participation in the study.

### Ethics approval

The study was approved by the National Taiwan University local ethics committee. The experiments and methods were conducted in accordance with applicable guidelines and regulations.

### Apparatus and stimuli

The Tobii X2 eye-tracking system was used to record the participants' eye movements at a sampling rate of 60 Hz. In this eye tracker, the fixation filter is Velocity-Threshold Identification (I-VT) with 0.4° of visual angle accuracy and a 30°/second velocity threshold^16^. The Tobii Studio 3.3.2 analysis package software was used for calibration, testing, and data analysis. The participant wore a pair of Sony 3D glasses integrated with an NVIDIA 3D vision IR Emitter and a Sony Bravia KDL-55W800B TV to perceive the stereoscopic 3D environment. The 3D TV screen was 123.4 cm $\times$ 76.4 cm. This experiment was performed on a high-speed computer with an Asus Intel® Core ™ i7-7700 CPU running at 3.60 GHz, 8 GB of RAM, and an NVIDIA GeForce GTX 1060 6 GB graphics card, and the monitor was a ViewSonic VA2448m-LED. The latency of the virtual system was reduced to the point where it had no effect on interaction performance.

**Figure 3:**
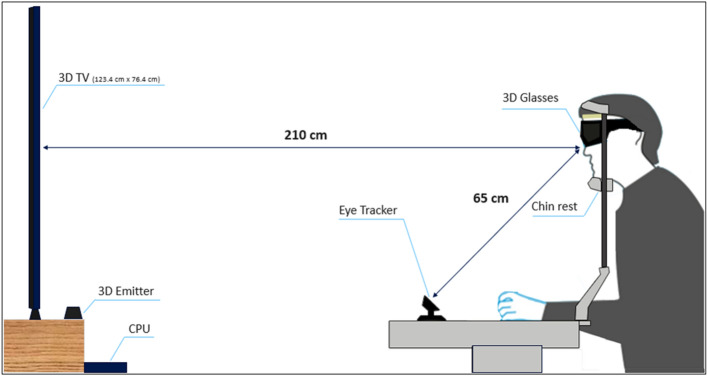
Illustration of the experimental setup.

An illustration of the experimental task is shown in Fig. 3. An excellent stereoscopic environment was created by creating a space of 3.6 m x 3 m x 2.5 m with black curtains, which blocked out all undesirable light. We followed the viewing distance requirements for 3D image perception proposed by the VQEG test plan^17^ and the Sony Bravia i-manual book^18^. Furthermore, Lin et al. (2019) concluded that the comfortable viewing distance for 55-inch 3D TVs is 3.93H (210 cm). Thus, the participants' eyes were 210 cm away from the 3D TV. The distance between the participant's eyes and the eye tracker in this study was approximately 65 cm (26"). This is the optimal distance between the participant's eye and the Tobii eye tracking device^19^. To ensure that the eye gaze data were accurate, chin rests were used to limit head movements and an adjustable chair was provided to ensure that each participant was in a comfortable position.

### Experiment procedures

All participants were required to pass the Tumbling E visual acuity test before the experiment. If their visual acuity was greater than 20/20, their visual acuity was optimal. Before the experiment, we calculated the parallax threshold to check the maximum stereo vision of each participant. The inter-pupillary distance (IPD) of those who qualified as adequate for the 3D vision used in this study was measured and the participants were asked to fill out a written consent form. After being informed of the experiment's goals and explanations, the participants volunteered to experience a virtual reality environment.

**Figure 4:**
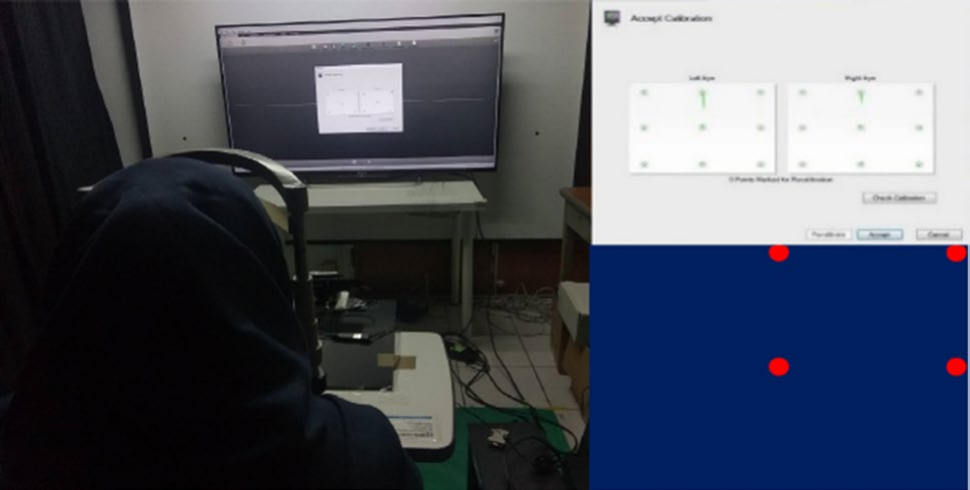
Illustration of a participant during the experiment.

The participants were directed to place their chins on a chin rest while wearing Sony 3D glasses (Fig. 4.). Prior to alteration of the parallax, the Tobii eye tracker was calibrated to ensure that it could detect the participant's eye movements. To capture the participant's eye gaze binocularly, a standard calibration setting from Tobii studio with nine points and medium speed was used by default. The participants were instructed to look at the red calibration points precisely until they disappeared. The calibration test makes it possible to determine whether the eye tracker is accurate and reliable. All test points were visualized with a red circle. By looking at the circles' calibration points, it is possible to determine if the calibration is accurate or if additional re-calibration is required^19^. If the participant's point was within the circle, the calibration was considered good and the experiment could proceed.

**Figure 5:**
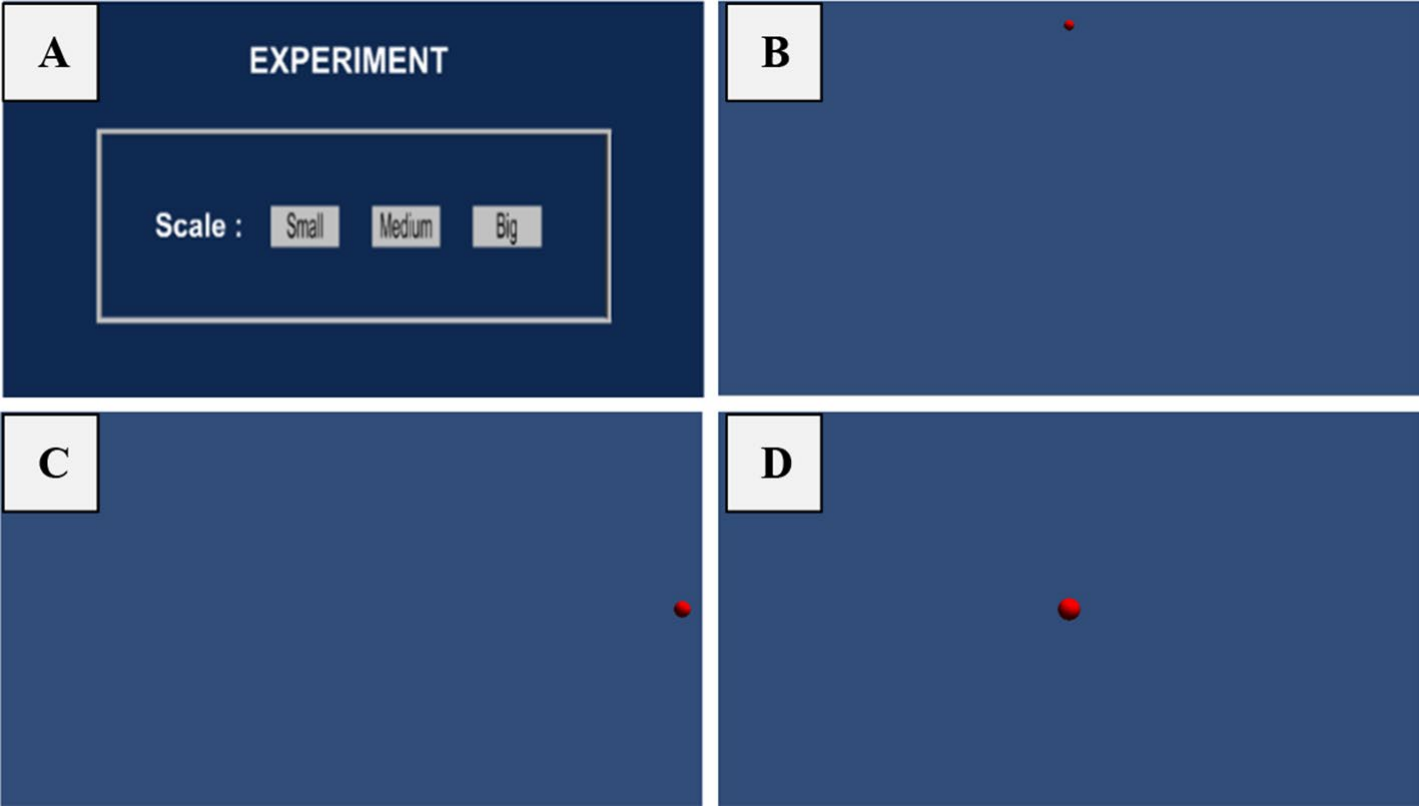
(**A**) Menu display (**B**) Object of small size in the middle top (**C**) Object of medium size in the middle right (**D**) Object of large size in the middle (all pictures in the same parallax).

The experiment took about 30 min. Four virtual spherical objects presented randomly in four positions in every scenario (3 different sizes and parallaxes). Participants were asked to look at the spherical object until it disappeared and then to look at the subsequent spherical object. In every trial, the Tobii system simultaneously tracked participants' eye gaze movements and fixation points. After completing the task in the first scenario, the participants continued to look at four spherical objects in the following scenario. The scenario consisted of 3 (parallaxes) x 3 (sizes) x 4 (positions) for each participant to complete. Therefore, every participant completed 36 trials. An illustration of the scenario is provided in Fig. 5.

### Experimental design

The experimental task is presented in Figs. 4 and 5. with stereoscopic targets projected in front of the 3D TV at various egocentric distances, positions, and sizes (on a frontal plane to the participants). The four spherical targets were displayed in red and in random order.

### Independent variables

This study had three independent variables: three parallax levels (target displayed at zero, 30 cm, and 60 cm parallax), three target sizes (small (1.9 cm), medium (2.9 cm), and large (3.9 cm) in diameter), and four object positions (top middle, top right, middle right, and middle). Using repeated-measures analysis of variance, the experiment was designed as a 3 (parallax) $\times$ 3 (size) $\times$ 4 (position) within-subject design; therefore, this study included 36 combinations within each subject.

### Dependent variables

In this study, the primary dependent variable was eye vergence angle. The vergence angle of the eye was measured by recording the eye gaze using data from the eye movement tracker. In general, the eye tracker's analysis software can determine an eye gaze position. When measuring gaze position, the projection of the gaze line onto the observed surface is taken into account, rather than the eye rotation angle (3D TV Screen). This means that the eye vergence angle will not be measured automatically. Additional computations must be performed to determine the vergence angle from raw eye-tracking data.

**Figure 6:**
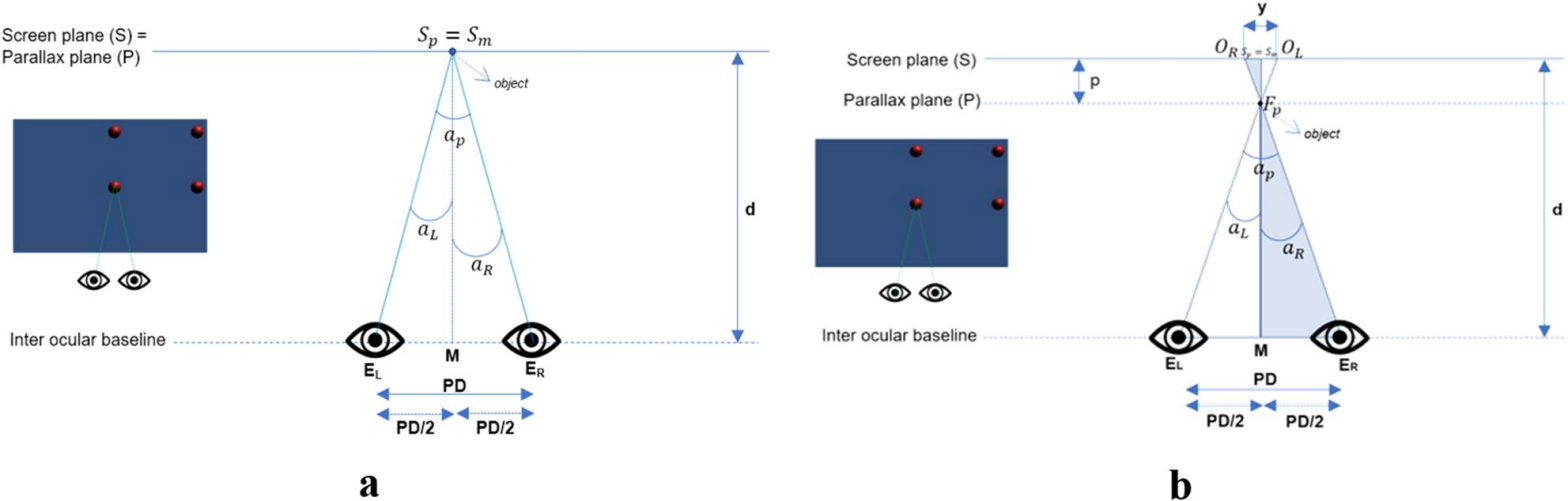
The virtual object in the middle for (**a**) zero parallax and (**b**) negative parallax.

As the participant looks at the 3D TV, the vergence angle is the angle between left and right gaze lines. The eye tracker provides data on the gaze line's projection; gaze position is represented in bidimensional coordinates relative to the surface of a 3D TV screen. To derive the eye vergence angle equation, we will consider the case in Fig. 6.

Where: E_R_: The right eye rotation center, E_L_: The left eye rotation center, PD : The distance between the E_R_ and E_L_ eye rotation centers, d: The distance between the screen plane and the inter ocular baseline, M: The midpoint between E_R_ and E_L,_${S}_{m}$: The orthogonal projection of the M on-screen plane corresponding to the screen's horizontal meridian, J: The distance between ${S}_{P}$ and ${S}_{m}$, ${O}_{R}$: The center of the object on the screen plane for the right eye, ${O}_{L}$: The center of the object on the screen plane for the left eye, y: The distance between ${O}_{R}$ and ${O}_{L}$, ${S}_{p}$: The orthogonal projection of ${F}_{p}$ (fixation point) on the screen plane, ${F}_{p}$: The fixation point, ${S}_{R}$: On-screen plane projection of the right eye line in the primary position, ${F}_{R}$: On-parallax plane projection of the right eye line of gaze in the primary position, ${a}_{P}$: Vergence angle, ${a}_{R}$: Right eye angle, ${a}_{L}$: Left eye angle, ${\mathrm{g}}_{L}$: Left eye gaze vector, ${\mathrm{g}}_{R}$: Right eye gaze vector.

We consider the case above to derive the eye vergence angle equation. Because the equations are based on the same functions in trigonometry, the equation can be derived to be:

1 $${a}_{p}={a}_{L}-{a}_{R}$$

2 $${a}_{R}=ArcTan\left[\frac{{\text{J}}-\frac{\text{PD}}{2}}{{\text{d}}-{\text{p}}}\right]$$

Considering the similar triangles ${F}_{p}$ M E_R_ and ${O}_{L}{S}_{p}{F}_{p}$, we can derive:

$$\frac{\text{p}}{\left(\frac{\text{y}}{2}\right)}=\frac{\left({\text{d}}-{\text{p}}\right)}{\left(\frac{\text{PD}}{2}\right)}$$

3 $${\text{p}}=\frac{{\text{d}}{\text{y}}}{{\text{PD}}+{\text{y}}}$$

**Figure 7:**
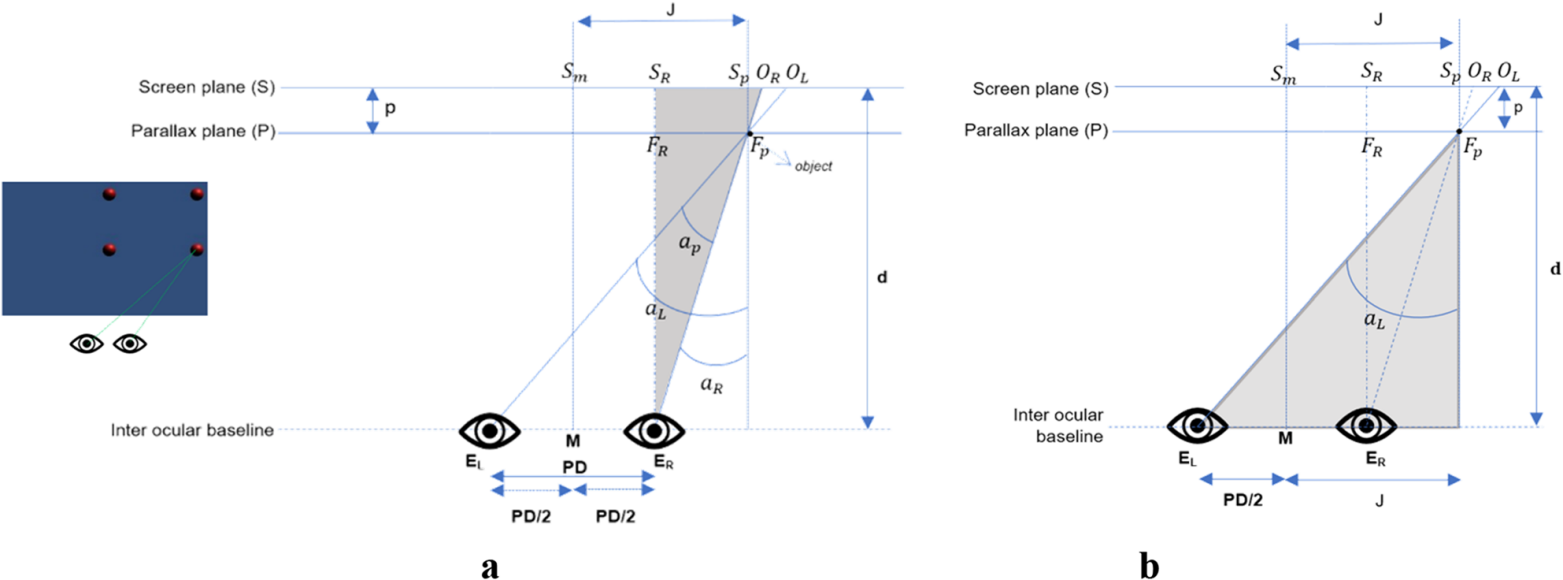
The virtual object in negative parallax and on the right.

The unknown value of ${\text{J}}$ can be determined based on Fig. 7a. From comparing the similar triangles ${S}_{R}$ E_R_ ${O}_{R}$ and *F*_*R*_E_R_ ${F}_{p}$, we can determine:

4 $${\text{J}}=\frac{\text{PD}}{2}+\overline{{S }_{R}{S}_{p}}$$

5 $$\frac{{\text{d}}-{\text{p}}}{\text{d}}=\frac{\overline{{F }_{R}{F}_{P}}}{\overline{{S }_{R}{O}_{R}}}$$

where $$\overline{{F }_{R}{F}_{p}}=\overline{{S }_{R}{S}_{p}}$$ :

6 $$\overline{{S }_{R}{S}_{p}}=\frac{\overline{{S }_{R}{O}_{R}}\left({\text{d}}-{\text{p}}\right)}{\text{d}}$$

Based on Fig. 7a., segment $\overline{{S }_{R}{O}_{R}}$ can be determined as

7 $$\overline{{S }_{R}{O}_{R}}={O}_{R}-\left({S}_{m}+\frac{\text{PD}}{2}\right)$$

Then Equation 7 can be substituted into Equation 6:

8 $$\overline{{S }_{R}{S}_{p}} =\frac{\left({O}_{R}-\left({S}_{m}+\frac{\text{PD}}{2}\right)\right)\left({\text{d}}-{\text{p}}\right)}{\text{d}}$$

where ${\text{J}}$ is computed as

9 $${\text{J}}=\frac{\text{PD}}{2}+\frac{\left({O}_{R}-\left({S}_{m}+\frac{\text{PD}}{2}\right)\right)\left({\text{d}}-{\text{p}}\right)}{\text{d}}$$

${\text{J}}$ is solved by Eq. (9) and ${\text{p}}$ by Eq. (3), and ${a}_{R}$ can be determined as

10 $${a}_{R}=ArcTan\left[\frac{\left({O}_{R}-\left({S}_{m}+\frac{\text{PD}}{2}\right)\right)}{\text{d}}\right]$$

To determine ${a}_{L}$, consider the grey triangle in Fig. 7b. and then substitute Eq. 9 and 3 into Eq. 11:

11 $${a}_{L}=ArcTan\left[\frac{\frac{\text{PD}}{2}+{\text{J}}}{{\text{d}}-{\text{p}}}\right]$$

12 $${a}_{L}=ArcTan\left[\frac{{\text{PD}}+\frac{\left({O}_{R}-\left({S}_{m}+\frac{\text{PD}}{2}\right)\right)\left({\text{d}}-\frac{{\text{d}}{\text{y}}}{{\text{PD}}+{\text{y}}}\right)}{\text{D}}}{{\text{d}}-\frac{{\text{d}}{\text{y}}}{{\text{PD}}+{\text{y}}}}\right]$$

For alternative equations for Eq. 12, we can use gaze vectors based on Fig. 7. We can find the 2D coordinates of the gaze vectors ${\mathrm{g}}_{L}$ and ${\mathrm{g}}_{R}$ as follows:

13 $${\mathrm{g}}_{L}=\left({O}_{L}-{S}_{m}+\frac{\text{PD}}{2},d\right)$$

14 $${\mathrm{g}}_{R}=\left({O}_{R}-{S}_{m}-\frac{\text{PD}}{2},d\right)$$

where ${O}_{L}$ = ${O}_{R}$  + ${\text{y}}$, thus, we can determine:

15 $${a}_{L}=ArcTan\left[\frac{{O}_{L}-{S}_{m}+\frac{\text{PD}}{2}}{\text{d}}\right]$$

Therefore, to compute eye vergence angle ${\alpha }_{p}$, the final step is to substitute Eqs. (10) and (12) with (1):

16 $${a}_{p}=ArcTan\left[\frac{{\text{PD}}+\frac{\left({O}_{R}-\left({S}_{m}+\frac{\text{PD}}{2}\right)\right)\left({\text{d}}-\frac{{\text{d}}{\text{y}}}{{\text{PD}}+{\text{y}}}\right)}{\text{d}}}{{\text{d}}-\frac{{\text{d}}{\text{y}}}{{\text{PD}}+{\text{y}}}}\right]-ArcTan\left[\frac{\left({O}_{R}-\left({S}_{m}+\frac{\text{PD}}{2}\right)\right)}{\text{d}}\right]$$

Alternatively, we can use scalar product from the gaze vectors ${\mathrm{g}}_{L}$ and ${\mathrm{g}}_{R}$ above as

17 $$ a_{p} = Arc{\text{Cos}} \left[ {\frac{{g_{L} \cdot g_{R} }}{{g_{L} g_{R} }}} \right] $$

18 $${a}_{p}=ArcCos\left[\frac{\left({O}_{L}-{S}_{m}+\frac{\text{PD}}{2}\right)\left({O}_{R}-{S}_{m}-\frac{\text{PD}}{2}\right)+{\text{d}}^{2}}{\sqrt{{\left({O}_{L}-{S}_{m}+\frac{\text{PD}}{2}\right)}^{2}}+{\text{d}}^{2}\sqrt{{\left({O}_{R}-{S}_{m}-\frac{\text{PD}}{2}\right)}^{2}}+{\text{d}}^{2}}\right]$$

This study described a set of equations with two relevant features. First, it considers a parameter rarely considered in software for data analysis, such as specific interpupillary distance. As a second consideration, the eccentricity of gaze position when looking at the central zone of a 3D TV screen or a peripheral area in relation to the observed plane center, is considered in the computation to avoid being overestimated in the vergence determination. Although, height differences exist in these experiments, but they can be neglected for the sought precision levels. Therefore, the two-dimensional approach outlined above is appropriate for this study.

The other dependent variables are signed error and accuracy. The signed error (SE) was calculated as the difference between the ratio of the gaze-based eye vergence angle and the theoretical eye vergence angle using Eq. (19)^12^. Positive values of signed error indicate vergence angle overshoot, negative values indicate undershoot, and zero indicates an exact match.

19 $$\mathrm{Signed\, error }(\mathrm{SE})\hspace{0.17em}=\frac{{a}_{p}\ gaze \,based}{{a}_{p}\ theoretical}-1$$

The accuracy determines how close the eye vergence angle is to the theoretical eye vergence angle based on eye gaze data collected by the eye tracker. In the beginning, the theoretical eye vergence angle was calculated using the values of each parameter. Following that, the eye vergence angle accuracy was calculated by the formula^20,21^:

20 $$\mathrm{Accuracy}\hspace{0.17em}=\left(1-\left|Signed \,Error\right|\right)$$

where ${a}_{p} gaze\, based$ represents the participant’s eye vergence angle as determined with data from the eye tracker, and ${a}_{p} theoretical$ represents the corresponding actual or reference eye vergence angle. 

## Data Availability

Data generated and/or analyzed during this research are available for reasonable requests from the corresponding author. Due to privacy and ethical concerns, the data are not publicly available.
